# Supporting the inclusion and retention of autistic students: Exploring teachers' and paraeducators' use of evidence-based practices in public elementary schools

**DOI:** 10.3389/fpsyt.2022.961219

**Published:** 2022-12-06

**Authors:** Jill Locke, Alyssa M. Hernandez, Mahima Joshi, Maria L. Hugh, Alice Bravo, Anthony Osuna, Michael David Pullmann

**Affiliations:** ^1^Department of Psychiatry and Behavioral Sciences, University of Washington, Seattle, WA, United States; ^2^Department of Special Education, University of Kansas, Lawrence, KS, United States

**Keywords:** autism, inclusion, paraeducator, educators, evidence-based practice

## Abstract

**Introduction:**

Educators in public schools are required to serve students in their least restrictive environment. While many evidence-based practices (EBPs), defined as practices and strategies shown by research to have meaningful effectson outcomes for autistic students are documented in the literature, less is known about EBP use among educators in public schools.

**Methods:**

Eighty-six general and special education teachers and para educators completed a survey about familiarity, training, and EBP use for included autistic children.

**Results:**

Across roles, educators reported familiarity (98.8%), use (97.7%), and training (83.7%) in reinforcement. They reported the least familiarity with behavioral momentum (29.1%), training in both video modeling and peer-mediated instruction and intervention (18.6%), and use of video modeling (14.0%). Follow-up interviews (*n* = 80) highlighted mixed understanding of EBP definitions and use.

**Discussion:**

Implications for inclusive education are discussed including autism-specific EBP training within pre-service teacher preparation programs.

One in 44 youth in the United States has autism spectrum disorder [ASD; (1)]. ASD is a pervasive developmental disorder characterized by deficits in social communication and the presence of restricted and/or repetitive behavior (2). As the primary setting in which autistic children receive services (3), schools are increasingly affected by this increased prevalence (4). Under the Individuals with Disabilities Education Act (5), schools are required to provide education in the least restrictive environment; thus, many autistic children are partially or fully included in general education settings (6). However, educators in these contexts have mixed familiarity, training, and varying degrees of support in their knowledge and use of evidence-based practices [EBPs; (7)], defined as practices and strategies shown by research to have meaningful effects on outcomes for autistic students (8). The types of EBPs most germane to inclusive settings and the extent to which general and special education teachers and paraeducators are trained to use EBPs is unknown (9, 10). Though EBP use is mandated in IDEA (11–13), research has shown that EBPs have not been successfully adopted, implemented, or sustained in these settings to support the inclusion and retention of autistic students (14–16). Understanding what interventions are used in these settings may provide a “road-map” for targeted efforts to improve the quality of training and supports for educators of autistic students (16, 17).

It is not clear which EBPs, if any, general and special education teachers and paraeducators are *familiar* with, commonly *trained* to use, or *use* to support inclusion of autistic students. Research improving the delivery of autism services in public schools has focused on training teachers of autistic children in self-contained settings (13, 18–20). Bond et al. (21) reviewed educational interventions for autistic children and found that a promising 59% of the studies were implemented by or involved teachers and paraeducators. While this is an encouraging finding, few of these studies were conducted in general education settings. Despite the growing number of autistic children in general education settings (22, 23), far less attention has been afforded to improving the instructional practices of general education teachers and paraeducators (24). It is critical not only to better understand teachers' practices but also explore the role of paraeducators in supporting teachers and included autistic children (25).

Inclusion alone may be necessary but not sufficient for improving outcomes for autistic children. Inclusion is the “fundamental concept that [autistic] children can and should be educated in the same setting as their [neurotypical] peers” [(26), p. 337]. The ways in which inclusion is facilitated in schools vary. Time in general education settings may be based on autism severity, where more impacted autistic children spend less time, and less impacted autistic children spend more time in general education (27). As expected, there is tremendous variability in the success of included autistic children (28, 29). Teachers and paraeducators may struggle to meet the range of intensive academic and developmental needs of autistic children in general education settings or rely on general teaching strategies that have little relevance to autistic children (30, 31). The complex and heterogeneous presentation of ASD symptoms may require specialized autism training that often is limited and unavailable in standard teacher preparation programs (32–37).

A growing number of EBPs can be used to include and retain autistic children in general education settings purposefully (38–40). In 2015, the National Professional Development Center on Autism Spectrum Disorder (NPDC) applied a rigorous set of criteria to classify 27 interventions as EBPs (38). In 2020, the NPDC updated their review finding 28 EBPs with some overlap of the previous EBPs (e.g., scripting and visual supports) and initial evidence qualifying as EBP for two new practices [Sensory Integration Therapy, and music-mediated intervention (40)]. While important, these lists do not ensure that teachers and paraeducators consistently receive training to deliver EBPs for autistic children in schools (41, 42). In fact, there is a large disconnect between best practice guidelines and actual practice (14, 43, 44).

For EBPs to be used, educators must be familiar with and trained in them. However, the extent to which general and special education teachers and paraeducators are familiar with and trained to use EBPs and the types of EBPs most germane to inclusive settings remains minimally explored (9, 10, 24, 45). Studies that have examined EBP use among educators have traditionally focused on teachers and not paraeducators. McNeill (7) found that educators' knowledge and use of EBPs for autistic students were closely related. Given that these same EBPs often are not successfully adopted, implemented, or sustained in public schools (15, 46), familiarity and knowledge should be explored to examine this chasm. Barry et al. (24) found that the majority of teachers received little initial teacher education training in autism and almost no continuous professional development before educating an autistic child. The lack of specific knowledge about the use of EBPs may impede implementation efforts to increase use, which could be addressed by more nuanced research.

The purpose of this study was to identify which EBPs general and special education teachers and paraeducators have heard of, been trained in, and use to include and retain autistic children in general education settings more meaningfully. Specifically, this study had the following research questions: (1) Which EBPs have teachers and paraeducators heard of? (2) Which EBPs for autistic children have general and special education teachers and paraeducators been trained to use to support inclusion? (3) Which EBPs do teachers and paraeducators use to support included autistic children? (4) What are the most frequently used EBPs to support the inclusion and retention of autistic children in general education classrooms and how do they differ by role? (5) How do teachers and paraeducators understand EBPs? An exploratory, mixed-methods design was used to obtain educator-reported EBP familiarity, training, and use. These data will allow us to understand usual care practices in schools to support the inclusion of autistic children in general education settings.

## Methods

### Participants and setting

A free web-based tool, https://thegeneralizer.org, was used to obtain a representative sample of schools based on selected school and district characteristics (47). The web-based tool allowed us to capture a broad range of perspectives on the use of EBPs in general education settings to achieve data saturation. The target sample was intended to infer to Washington State schools from Kindergarten-5th grade (K-5), with representative balance based on school characteristics (size, % free and reduced lunch, gender, race, urbanicity/rurality) and district characteristics (number of schools, % English Language Learners, % language at home, English only, and urbanicity/rurality). In total, 56 schools in 28 districts were invited to participate in the study. Approximately 126 interest forms from school educators were received. Educators were screened per established criteria: (1) identified as a general education teacher, special education teacher, or paraeducator; (2) supported an autistic student who participates in a general education setting at least 15 min per day; and (3) worked in a Washington state public elementary school. One-hundred and four interested educators who met criteria were sent the study survey *via* a Qualtrics-generated hyperlink. Eighty-six educators from 50 schools from 24 districts in WA state completed surveys. The generalizability index is a score between 0 and 1, with 1 indicating that the sample is exactly the same as the population on the characteristics described above, and a 0 indicating that the sample and population share no common features (48). The final recruited sample achieved a generalizability index score of 0.92 for Washington State (considered very high), and 0.78 for the US population of schools (considered high) in regard to the characteristics described above.

### Demographics

Table 1 presents descriptive information on enrolled participants. A total of 86 educators participated across three roles: general educators (*n* = 27), special educators (*n* = 31), and paraeducators (*n* = 28) who supported at least one autistic student in an inclusive general education classroom; 80 of these participants completed follow-up interviews. Most educators were between ages 35–44 (*n* = 30, %=34.9) and had an average of 6.4 years in their current position (*SD* = 4.8). Sex distribution was 91.9% female (*n* = 79), 8.1% male (*n* = 7). One paraeducator identified as Latinx (1.2% of entire sample). Educators self-identified as Asian (3.5%, *n* = 3), white (86.2%, *n* = 74), or multiracial (9.3%, *n* = 8). No participants identified exclusively as Black, Native American, or Hawaiian/Pacific Islander. There were no significant differences among roles based on sex, age, ethnicity, or race.

**Table 1:** Demographics.

	**Entire sample**		**General education teacher**		**Special education teacher**		**Paraeducators**		***p*-value**
	**N/M**	**%/SD**	**N/M**	**%/SD**	**N/M**	**%/SD**	**N/M**	**%/SD**	
N	86	100.0	27	31.4	31	36.0	28	32.6	
**Sex**
Female	79	91.9	25	92.6	30	96.8	24	85.7	0.296
Male	7	8.1	2	7.4	1	3.2	4	14.3	
**Age**
25–34	22	25.6	6	22.2	12	38.7	4	14.3	0.194
35–44	30	34.9	11	40.7	7	22.6	12	42.9	
45–44	19	22.1	5	18.5	9	29.0	5	17.9	
55–64	14	16.3	4	14.8	3	9.7	7	25.0	
65–74	1	1.2	1	3.7	0	0	0	0	
**Ethnicity**
Latinx	1	1.2	0	0	0	0	1	3.6	0.351
**Race**
White	74	86.2	22	81.5	30	96.8	22	81.5	0.292
Asian	3	3.5	2	7.4	0	0	1	3.7	
Black	0	0	0	0	0	0	0	0	
Native American	0	0	0	0	0	0	0	0	
Hawaiian/Pacific Islander	0	0	0	0	0	0	0	0	
Multiracial^a^	8	9.3	3	11.1	1	3.2	4	14.8	
**Education**
High school diploma	7	8.1	0	0	0	0	7	25.0	< 0.001
Associate degree	5	5.8	0	0	0	0	5	17.9	
Bachelor's degree	28	32.6	4	14.8	10	32.3	14	50.0	
Master's degree	46	53.5	23	85.2	21	67.7	2	7.1	
**Years in current position**	6.4	4.8	8.0	5.3	4.8	4.5	6.7	4.3	0.047
**Certification** ^**b**^
General education	57	66.3	27	100.0	22	71.0	8	28.6	< 0.001
Special education	43	50.0	6	22.2	31	100	6	21.4	< 0.001
Social work	3	3.5	0	0	0	0	3	10.7	0.04
BCBA or BCaBA	2	2.3	0	0	2	6.5	1	3.6	0.163
Other	28	32.6	9	33.3	5	16.1	14	50.0	0.021
**Number of certifications received**	1.5	0.66	1.56	0.70	1.94	0.63	1.11	0.31	< 0.001
**Classroom model^b^**
Co-taught inclusive	13	15.1	2	7.4	6	19.4	5	17.9	0.397
Special education inclusive	10	11.6	0	0	4	12.9	6	21.4	0.045
Self-contained	35	40.7	1	3.7	19	61.3	15	53.6	< 0.001
N	86	100.0	27	31.4	31	36.0	28	32.6	
General education inclusive	57	66.3	24	88.9	16	51.6	17	60.7	0.008
Other	8	9.3	2	7.4	3	9.7	3	10.7	0.911
**Number of classroom models used**	1.4	0.74	1.07	0.267	1.55	0.810	1.64	0.870	0.008

a Multiracial breakdown: White, Black or African American, and American Indian/Alaskan Native; White, Asian, and Pacific Islander; White and Asian; White and American Indian/Alaskan Native; White and Black or African American. Differences among groups tested with ANOVAs and crosstabulations with chi-square tests.

b Certification and Classroom Model sections add to >100%.

All statistical testing was done using crosstabulations with chi-square tests.

### Procedures

The University of Washington's Institutional Review Boards (IRB) approved the study. When applicable, school district IRB approval was sought. Eight school district IRB applications were submitted and five school district IRB approvals were obtained. We contacted school district officials to obtain a list of elementary schools that have enrolled autistic children who are partially or fully included in a general education classroom and then emailed the school principal. Approximately 358 school principals were emailed. Subsequently, we distributed all recruitment materials (e.g., flyers, consent materials) to general and special education teachers and paraeducators. Before participation, the research team provided a full description to all participants of study procedures and activities included in study participation. Upon consent, participants were asked to complete a modified Autism Treatment Survey (ATS; 13) *via* an online survey. After completion, all participants were invited to an audio-recorded semi-structured interview (30–45 min) at a convenient time for the participant (*via* Zoom). Of the 86 participants, 80 agreed to do a follow-up interview. As an incentive for their participation, all participants were offered a $40 gift card, and their schools received a resource kit of materials to use with autistic children (e.g., Velcro, visual timer, visual supports, sensory toys, and fidgets, etc.).

### Measures

#### Modified Autism Treatment Survey [ATS]

The ATS was designed to measure teachers' frequency of use and training experiences with a comprehensive list of EBPs for autistic children (14). Given the increased research evaluating the efficacy of practices since the origination of the ATS, we have updated the ATS as follows: (a) we included practices that have been identified as evidence-based for autistic students since its publication according to the NPDC (40); (b) we separated evidence-based *multicomponent practices* (e.g., peer-based intervention) from *strategies* (e.g., modeling); (c) we used updated practice definitions that align with the NPDC that have been validated *via* cognitive interviewing in more recent research (49); and (d) we break down components of the implementation strategy—training—to inform later implementation support development (i.e., who provided the training, what type of training). In addition, we added a question about participants' familiarity with each practice with a dichotomous question that asked whether they had heard of the practice (49, 50).

Addressing calls for more specific investigations into subtypes of EBPs, we took a novel approach to autism EBP exploration by attending to both multicomponent practices and strategies (51). To develop a set of multicomponent practices and strategies that are used to support meaningful inclusion and retention of autistic children in general education settings, we used explicit criteria for exclusion of practices from the NPDC review—practices could not: (1) be assessments (e.g., functional behavior assessment); (2) implemented only outside of schools (e.g., parent-implemented); or (3) require certification (e.g., Pivotal Response Training, Sensory Integration Therapy). We reviewed these criteria and the final set of evidence-based multi-component practices and strategies with an autism intervention expert. Twenty-one EBPs from the 28 listed in the NPDC were included. Multicomponent EBPs included: social narratives, direct instruction, antecedent-based interventions, social skills training, self-management, functional communication training, discrete trial training, peer-mediated instruction and intervention, and naturalistic intervention. Evidence-based strategies included: reinforcement, modeling, task analysis, visual supports, prompting procedures, augmentative and alternative communication, extinction, response interruption/redirection, time delay, technology-aided instruction and intervention, video modeling, and behavioral momentum intervention. Distinguishing multicomponent practice and strategy was necessary to better understand the possible limited use of multicomponent practices rather than excluding these if participants report frequent use of strategies only (7, 43).

### Semi-structured interview

To complement the modified ATS and more thoroughly explore EBP use to support and retain autistic children in general education settings, we developed a systematic and comprehensive interview guide with questions that explored what the terms “evidence-based practice” and “evidence-based strategy” meant to participants and how they used the identified EBPs from their survey responses. Questions were carefully constructed to elicit clear information without assigning valence to EBP use. We asked each participant about two to four EBPs from their survey. To achieve saturation across practices, strategies, training, and frequency of use, we selected each participant's EBPs based on the following decision-guide: one practice that they had been trained in but did not use, one practice and one strategy that they used frequently regardless of training, and one practice or strategy that was rarely used regardless of training.

Four research team members conducted interviews. Interviews were conducted *via* Zoom, and audio and visual media were recorded following the participant's consent. Interviewers recorded field notes to be referenced for documentation, recorded emerging themes, and uploaded audio media to Rev.com for transcription. Interviewers reviewed transcripts for accuracy and to confirm validity to audio recording, provide clarity around inaudible segments, and remove identifiable transcript text (e.g., name of school, district, city, etc.).

### Data analysis

Crosstabulations with chi-square tests were used to compare educator roles on demographic and descriptive information. ANOVAs were computed to compare roles on the mean total number of different multicomponent practices and strategies the participants were familiar with, trained to use, or used. Due to the lack of homogeneity of variance, Ganes-Howell *post-hoc* tests were used to test between group differences for the number of multicomponent practices/strategies in which participants were familiar. Tukey's Honestly Significant Difference tests were used for multicomponent practices/strategies trained in and used. We descriptively analyzed the modified ATS by computing percentages of those who were familiar with the practice/strategy; who were trained to use the practice/strategy, and those who used the practice/strategy in the last 2 years.

For qualitative data, the principal investigator and three research team members independently coded an initial set of transcripts to identify codes and met as a group to discuss recurring codes. We developed a codebook with operational definitions and examples of when to use and not use the code; an integrated approach to coding as certain codes were conceptualized during the interview guide development (i.e., deductive approach) and other codes were developed through a close reading of the initial set of transcripts [i.e., inductive approach; (52)]. The coding scheme was refined throughout the data analytic process (52). Following the development of a stable codebook, three team members coded ten randomly selected transcripts to train and achieve inter-rater reliability above 90%. After achieving initial reliability of 95.1%, coders interpedently coded the remaining transcripts, overlapping on 20% of randomly selected transcripts to maintain interrater reliability; drift reliability was 95.5%. To capture data complexity, avoid errors, reduce groupthink, and circumvent some researcher biases, a consensus process was used in which all reviewers independently coded reliability transcripts and met to compare their coding to arrive at consensus judgments through open dialogue (53–55). We used the approach of inductive thematic saturation to determine when there are no new emerging codes or themes, meaning data saturation has been reached (56).

## Results

### Modified Autism Treatment Survey [ATS; (14)]

**Figure 1:**
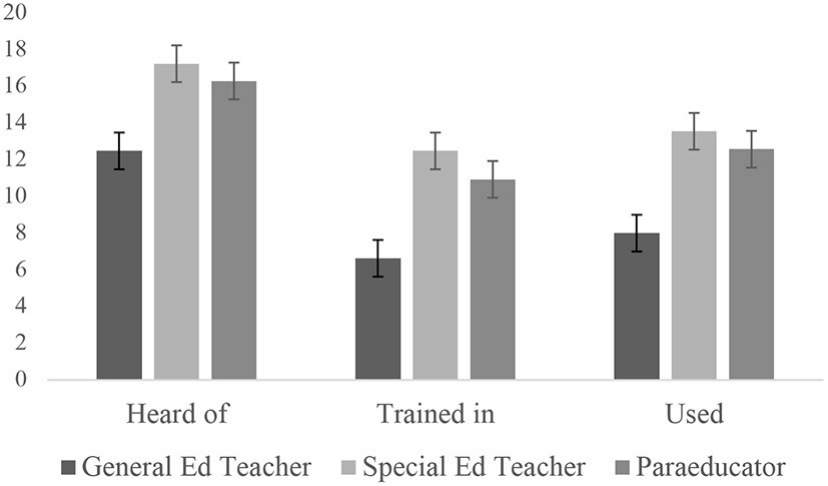
Number of different practices educators were familiar with, trained in, or used, with standard deviation whiskers.

Quantitative data were collected from 86 educators. Educator roles significantly differed by the mean number of different multicomponent practices and strategies in which they were familiar (*F* = 12.1, *p* < 0.001), trained in (*F* = 9.4, *p* < 0.001), or used (*F* = 17.5, *p* < 0.001). *Post-hoc* testing revealed that general education teachers had significantly fewer multicomponent practices and strategies in which they were familiar, trained in, and used compared to the other two roles (see Figure 1). There were no significant differences between special education teachers and paraeducators on any variables.

### Familiar multicomponent practices and strategies

Across educator roles, reinforcement (98.8%), modeling (97.7%), and task analysis (94.2%) were the most familiar multicomponent practices or strategies, and behavioral momentum intervention (29.1%), video modeling (44.2%), and naturalistic intervention (44.2%) were the least familiar multicomponent practices or strategies (Table 2).

**Table 2:** Multicomponent practice and strategies heard of (ranked by entire sample).

	**Heard of multicomponent practices and strategies**							
	**Entire sample (*****N*** = **86)**		**General educators (*****N*** = **27)**		**Special educators (*****N*** = **31)**		**Paraeducators (*****N*** = **28)**	
	***n***	**% of *N***	**n**	**% of *N***	***n***	**% of *N***	***n***	**% of *N***
Reinforcement	85	98.8	26	96.3	31	100.0	28	100.0
Modeling	84	97.7	25	92.6	31	100.0	28	100.0
Task analysis	81	94.2	23	85.2	31	100.0	27	96.4
Visual supports	80	93.0	25	92.6	31	100.0	24	85.7
Social narratives	78	90.7	20	74.1	31	100.0	27	96.4
Direct instruction	77	89.5	25	92.6	30	96.8	22	78.6
Antecedent-based interventions	76	88.4	21	77.8	28	90.3	27	96.4
Social skills training	75	87.2	21	77.8	29	93.5	25	89.3
Self-management	70	81.4	18	66.7	30	96.8	22	78.6
Prompting procedures	69	80.2	16	59.3	27	87.1	26	92.9
Augmentative and alternative communication	68	79.1	18	66.7	28	90.3	22	78.6
Extinction	64	74.4	14	51.9	28	90.3	22	78.6
Functional communication training	63	73.3	17	63.0	23	74.2	23	82.1
Response interruption/redirection	62	72.1	14	51.9	23	74.2	25	89.3
Time delay	55	64.0	13	48.1	24	77.4	18	64.3
Discrete trial teaching	47	54.7	5	18.5	26	83.9	16	57.1
Peer-mediated instruction and intervention	42	48.8	8	29.6	17	54.8	17	60.7
Technology-aided instruction and intervention	39	45.3	13	48.1	10	32.3	16	57.1
Naturalistic intervention	38	44.2	6	22.2	16	51.6	16	57.1
Video modeling	38	44.2	7	25.9	21	67.7	10	35.7
Behavioral momentum intervention	25	29.1	1	3.7	12	38.7	12	42.9
**Total** ***N***	**86**		**27**		**31**		**28**	

### Familiar multicomponent practices and strategies by role

General educators were most familiar with reinforcement (96.3%), direct instruction (92.6%), modeling (92.6%), and visual supports (92.6%); and least familiar with behavioral momentum intervention (3.7%), discrete trial teaching (18.5%), and naturalistic intervention (22.2%). Special educators were most familiar with social narratives (100.0%), reinforcement (100.0%), modeling (100.0%), task analysis (100.0%) and visual supports (100.0%) and least familiar with technology-aided instruction and intervention (32.3%), behavioral momentum intervention (38.7%), naturalistic intervention (51.6%). Paraeducators were most familiar with reinforcement (100.0%), modeling (100.0%), task analysis (96.4%), social narratives (96.4%), and antecedent-based interventions (96.4%) and least familiar with video modeling (35.7%), behavioral momentum intervention (42.9%), technology-aided instruction and intervention (57.1%), discrete trial teaching (57.1%), and naturalistic intervention (57.1%).

### Training in multicomponent practices and strategies

As shown in Table 3, educators were most frequently trained to use reinforcement (83.7%), modeling (79.1%), and visual supports (74.4%) and least frequently trained to use video modeling (18.6%), peer-mediated instruction and intervention (18.6%), and behavioral momentum intervention (19.8%).

**Table 3:** Multicomponent practice and strategies training (ranked by entire sample).

	**Training in multicomponent practices and strategies**							
	**Entire sample (*N =* 86)**		**General educators (*N =* 27)**		**Special educators (*N =* 31)**		**Paraeducators (N = 28)**	
	***n***	**% of *N***	***n***	**% of *N***	***n***	**% of *N***	**n**	**% of *N***
Reinforcement	72	83.7	21	77.8	29	93.5	22	78.6
Modeling	68	79.1	19	70.4	28	90.3	21	75.0
Visual supports	64	74.4	18	66.7	27	87.1	19	67.9
Task analysis	63	73.3	17	63.0	26	83.9	20	71.4
Prompting procedures	55	64.0	9	33.3	25	80.6	21	75.0
Direct instruction	52	60.5	13	48.1	27	87.1	12	42.9
Antecedent-based interventions	49	57.0	9	33.3	20	64.5	20	71.4
Extinction	49	57.0	6	22.2	25	80.6	18	64.3
Functional communication training	42	48.8	10	37.0	17	54.8	15	53.6
Self-management	40	46.5	9	33.3	18	58.1	13	46.4
Social narratives	40	46.5	8	29.6	15	48.4	17	60.7
Social skills training	40	46.5	9	33.3	18	58.1	13	46.4
Response interruption/redirection	38	44.2	7	25.9	13	41.9	18	64.3
Time delay	38	44.2	6	22.2	19	61.3	13	46.4
Augmentative and alternative communication	36	41.9	5	18.5	18	58.1	13	46.4
Discrete trial teaching	30	34.9	1	3.7	18	58.1	11	39.3
Naturalistic intervention	22	25.6	3	11.1	10	32.3	9	32.1
Technology-aided instruction and intervention	18	20.9	3	11.1	6	19.4	9	32.1
Behavioral momentum intervention	17	19.8	0	-	9	29.0	8	28.6
Peer-mediated instruction and intervention	16	18.6	3	11.1	4	12.9	9	32.1
Video modeling	16	18.6	3	11.1	10	32.3	3	10.7
**Total** ***N***	**86**		**27**		**31**		**28**	

### Training in multicomponent practices and strategies by role

General educators were most frequently trained to use reinforcement (77.8%), modeling (70.4%), and visual supports (66.7%) and least frequently trained to use behavioral momentum intervention (0%), discrete trial teaching (3.7%), and video modeling (11.1%). Special educators were most frequently trained to use reinforcement (93.5%), modeling (90.3%), and visual supports (87.1%) and least frequently trained to use peer-mediated instruction and intervention (12.9%), technology-aided instruction and intervention (19.4%), and behavioral momentum intervention (29.0%). Paraeducators were most frequently trained to use reinforcement (78.6%), modeling (75.0%), and prompting procedures (75.0%), and least frequently trained to use video modeling (10.7%), behavioral momentum intervention (28.6%), peer-mediated instruction and intervention (32.1%), technology-aided instruction and intervention (32.1%), and naturalistic intervention (32.1%).

### Use of multicomponent practices and strategies

As shown in Table 4, across educator roles and practice types (multicomponent or strategy), reinforcement (97.7%), modeling (94.2%), and visual supports (86.0%) were the most often used. Video modeling (14.0%), technology-aided instruction and intervention (17.4%), and behavioral momentum intervention (19.8%) were the least used. On a scale from 1 (rarely used) to 4 (very often used), the most frequently used (when only including those participants who used it to compute the mean score) were reinforcement (*M* = 3.61, *SD* = 0.58), modeling (*M* = 3.54, *SD* = 0.63), and prompting procedures (*M* = 3.49, *SD* = 0.63), and the least frequently used were video modeling (*M* = 2.25, *SD* = 0.75), peer-mediated instruction and intervention (*M* = 2.50, *SD* = 0.91), and extinction (*M* = 2.74, *SD* = 0.86).

**Table 4:** Multicomponent practice and strategies use (ranked by entire sample).

	**Use of multicomponent practices & strategies**															
	**Entire sample (*N =* 86)**				**General educators (*N =* 27)**				**Special educators (** ***N =* 31)**				**Paraeducators (*N =* 28)**			
	**Use**		**Frequency**		**Use**		**Frequency**		**Use**		**Frequency**		**Use**		**Frequency**	
	***n***	**% of *N***	**M of *n***	**SD**	***n***	**% of *N***	**M of *n***	**SD**	***n***	**% of *N***	**M of *n***	**SD**	***n***	**% of *N***	**M of *n***	**SD**
Reinforcement	84	97.7	3.61	0.58	25	92.6	3.60	0.65	31	100.0	3.77	0.43	28	100.0	3.43	0.63
Modeling	81	94.2	3.54	0.63	24	88.9	3.63	0.50	31	100.0	3.71	0.46	26	92.9	3.27	0.83
Visual supports	74	86.0	3.43	0.70	20	74.1	3.50	0.69	31	100.0	3.58	0.62	23	82.1	3.17	0.78
Antecedent-based interventions	66	76.7	3.09	0.82	18	66.7	3.06	0.80	25	80.6	3.16	0.75	23	82.1	3.04	0.93
Task analysis	65	75.6	3.34	0.71	15	55.6	3.60	0.51	28	90.3	3.32	0.72	22	78.6	3.18	0.80
Social narratives	63	73.3	2.83	0.98	13	48.1	2.46	1.13	29	93.5	2.93	0.96	21	75.0	2.90	0.89
Social skills training	62	72.1	3.19	0.88	16	59.3	2.56	0.73	26	83.9	3.58	0.70	20	71.4	3.20	0.95
Direct instruction	58	67.4	3.10	0.89	14	51.9	2.86	0.95	27	87.1	3.37	0.79	17	60.7	2.88	0.93
Prompting procedures	57	66.3	3.49	0.63	11	40.7	3.27	0.65	26	83.9	3.58	0.58	20	71.4	3.50	0.69
Functional communication training	48	55.8	3.06	0.78	12	44.4	3.17	0.72	17	54.8	3.12	0.70	19	67.9	2.95	0.91
Self-management	48	55.8	3.00	0.83	12	44.4	2.75	0.97	21	67.7	3.05	0.81	15	53.6	3.13	0.74
Extinction	46	53.5	2.74	0.86	7	25.9	2.57	0.98	22	71.0	2.86	0.56	17	60.7	2.65	1.12
Response interruption/redirection	42	48.8	3.05	0.85	6	22.2	2.83	0.75	16	51.6	3.00	0.89	20	71.4	3.15	0.88
Augmentative and alternative communication	41	47.7	2.76	0.92	9	33.3	2.78	0.83	17	54.8	2.65	1.17	15	53.6	2.87	0.64
Time delay	31	36.0	2.97	0.66	2	7.4	3.00	0.00	17	54.8	2.88	0.70	12	42.9	3.08	0.67
Discrete trial teaching	29	33.7	2.93	1.03	1	3.7	2.00	–	17	54.8	3.06	0.90	11	39.3	2.82	1.25
Naturalistic intervention	25	29.1	2.88	0.93	2	7.4	2.50	0.71	12	38.7	3.00	1.04	11	39.3	2.82	0.87
Peer-mediated instruction and intervention	22	25.6	2.50	0.91	5	18.5	2.40	0.55	5	16.1	2.60	1.14	12	42.9	2.50	1.00
Behavioral momentum intervention	17	19.8	2.76	1.09	0	–	–	–	9	29.0	2.78	1.09	8	28.6	2.75	1.17
Technology-aided instruction and intervention	15	17.4	3.07	0.96	3	11.1	2.67	1.16	5	16.1	3.40	0.89	7	25.0	3.00	1.00
Video modeling	12	14.0	2.25	0.75	1	3.7	2.00	–	7	22.6	2.29	0.76	4	14.3	2.25	0.96
**Total** ***N***	**86**				**27**				**31**				**28**			

“Use” means used in last 2 years; Frequency scale: “1”= rarely,“4”= Very often.

### Use of multicomponent practices and strategies by role

Among general educators, the most used multicomponent practices or strategies to facilitate inclusion of an autistic student were reinforcement (92.6%), modeling (88.9%), and visual supports (74.1%); behavioral momentum intervention (0%), discrete trial teaching (3.7%), and video modeling (3.7%) were the least used. On a scale from 1 (rarely used) to 4 (very often used), the most frequently used were modeling (*M* = 3.63, *SD* = 0.50), reinforcement (*M* = 3.60, *SD* = 0.65), and task analysis (*M* = 3.60, *SD* = 0.51). The least frequently used were video modeling, with one participant reporting use, (*M* = 2.00, *SD* = –); discrete trial teaching, with one participant reporting use (*M* = 2.00, *SD* = –); and peer-mediated instruction and intervention (*M* = 2.40, *SD* = 0.55).

Among special educators, the most used multicomponent practices or strategies to facilitate inclusion of an autistic student were reinforcement (100.0%), modeling (100.0%), and visual supports (100.0%) and the least used were peer-mediated instruction and intervention (16.1%), technology-aided instruction and intervention (16.1%), and visual modeling (22.6%). On a scale from 1 (rarely used) to 4 (very often used), of special educators who used a multicomponent practice or strategy, the most frequently used were reinforcement (*M* = 3.77, *SD* = 0.43), modeling (*M* = 3.71, *SD* = 0.46), visual supports (*M* = 3.58, *SD* = 0.62), social skills training (*M* = 3.58, *SD* = 0.70), and prompting procedure (*M* = 3.58, *SD* = 0.58). The least frequently used were video modeling (*M* = 2.29, *SD* = 0.76), peer-mediated instruction and intervention (*M* = 2.60, *SD* = 1.14), and augmentative and alternative communication (*M* = 2.65, *SD* = 1.17).

Among paraeducators, the most used multicomponent practices or strategies to facilitate inclusion of an autistic student were reinforcement (100.0%), modeling (92.9%), and antecedent-based interventions (82.1%) and the least used were video modeling (14.3%), technology-aided instruction and intervention (25.0%), and behavioral momentum intervention (28.6%). On a scale from 1 (rarely used) to 4 (very often used), the most frequently used multicomponent practices or strategies were prompting procedure (*M* = 3.50, *SD* = 0.69), reinforcement (*M* = 3.43, *SD* = 0.63), and modeling (*M* = 3.27, *SD* = 0.83). The least frequently used were video modeling (*M* = 2.25, *SD* = 0.96), peer-mediated instruction and intervention (*M* = 2.50, *SD* = 1.00), and extinction (*M* = 2.65, *SD* = 1.12).

### Semi-structured interview

Qualitative data were collected from 80 educators (*n* = 26 general education teachers, *n* = 30 special education teachers, and *n* = 24 paraeducators); six participants did not respond to the invitation or declined. Almost all educators believed that the term “EBP” meant that there is evidence (e.g., from their own experiences, others' work, and research) backing-up the effectiveness of the practices or strategies. Most also believed that EBPs need to be proven successful through replication in different settings, undergo “multiple trial and error”, and “peer review”. Some educators also mentioned “to get the desired effects, there needs to be data collection” and continuous effort to fit what individual students need. There were few educators who could not define EBPs or could only describe how they might use an EBP more broadly.

When asked about specific EBPs that educators said they used in their surveys, many educators had to be reminded what the specific EBP meant. Several participants asked the interviewer to provide the definition of that named EBP. Most educators could describe the components of the specific practice or strategy that was asked (e.g., steps that they use in the classroom with the student) but could not name the EBP. More educators asked for the definition (coded 50 times) compared to educators who knew what the term was and its definition (coded 19 times). There also were a few instances where the educators provided the wrong definition for a specific EBP they described. For example, one participant defined reinforcement when asked about antecedent-based intervention, “When I hear [Antecedent-Based Intervention], I'm thinking in my head, okay, so this is like a behavior chart where I'm reinforcing behaviors that I want and not reinforcing the behaviors that I don't, and then re-teaching new behaviors…”.

Almost all educators described how they used or applied specific EBPs with autistic students. The majority said they used additional school-based programs such as Second Steps or Edmark to complement their EBP use. Many participants also mentioned combining multiple EBPs together (e.g., prompting and visual supports, direct instruction and social skills training) to help their autistic students. There were a few educators who described the use of specific EBPs incorrectly–the most frequent misapplication was naturalistic intervention (e.g., 25% of the time naturalistic intervention was described did not match the definition in the literature).

## Discussion

The heterogeneity of autism symptoms poses a tremendous challenge in providing targeted, individualized, and meaningful EBPs to autistic children in public schools—no single EBP addresses the needs of all autistic children (8), which makes it almost impossible for educators to become familiar, receive training, and develop expertise to use a multitude of EBPs to support various autistic children in their care. Despite the catalog of promising, emerging, and established EBPs in the NPDC (40), it is still unclear which EBPs are used in elementary schools to support the inclusion and retention of autistic children in their least restrictive environment. This study is one of the first to prospectively understand the number and type of EBPs that elementary school general and special education teachers and paraeducators were familiar, trained in, and used to support autistic children in inclusive settings.

Overall, we found that educators were most familiar with and trained in reinforcement, modeling, task analysis, and visual supports but reinforcement, modeling, visual supports, and antecedent-based interventions were the most frequently used EBPs to support the inclusion and retention of autistic children in general education classrooms. We also found significant differences by educator role in that general education teachers had significantly less familiarity, training in EBPs, and use compared to special education teachers and paraeducators. Qualitative data explored nuanced differences in educators' understanding of EBPs and uncovered a mixed understanding of specific EBPs and their unique application to autistic students. The results of this study have important implications on how public schools consider and support EBP use among general and special education teachers and paraeducators for included autistic children.

Not surprisingly, all four EBPs (reinforcement, modeling, task analysis, and visual supports) that educators were most familiar with and trained in were evidence-based strategies. Only one EBP, antecedent-based intervention, was classified as a multicomponent practice. Although reinforcement, modeling, task analysis, and visual supports often are embedded steps in many of the queried multicomponent practices, these findings point to the challenges around EBP use of more “complex” interventions that comprise multiple strategies and components (51, 57). Multicomponent practices may be “harder” and more challenging for educators to implement, given their intervention complexity. One critical aspect of intervention complexity is that complex EBPs may require higher interventionist training and skill (57), a known barrier to implementing autism EBPs in school settings (28, 58). Interestingly, while these EBPs address ancillary behaviors related to the education of autistic children, they do not specifically address the core symptoms (e.g., social interaction and communication) underlying autism. Multicomponent practices with a strong evidence-base supporting social interaction and communication such as naturalistic intervention and peer-mediated instruction and intervention were infrequently used, despite evidence that educators can effectively implement these (59). Efforts to improve familiarity, training of targeted EBPs, and use to support specific outcomes such as social behaviors may be warranted to cultivate a more conducive environment for inclusion of autistic children.

The results also indicated that general education teachers had significantly less familiarity, training in EBPs, and use than their special education counterparts. These findings suggest that general education teachers may need more exposure to and training in autism-specific EBPs given the increased prevalence and high likelihood that autistic children will be included in their classrooms. There are several avenues in which this can occur. First, standard teacher preparation programs should consider requiring more specialized autism training (e.g., coursework, practicum, rotations, etc.) during educators' pre-service learning to build familiarity, practice, and gain mastery in EBP use (49, 60). Second, school districts and school buildings should prioritize in-service training or professional development opportunities to continuously learn new EBPs to support autistic children. Community-academic partnerships may be one avenue to support the training of providers to fidelity in autism EBPs (61, 62). Lastly, while most schools have adopted teams that comprise general and special educators and specialists (e.g., counselors, psychologists, speech therapists, etc.) to support autistic children, there may be some utility in protecting shared planning or meeting time to discuss supports for autistic children, monitor and track progress, or problem-solve around EBP implementation.

While it is promising that most educators understood what the term “EBP” meant, the qualitative data suggested there is some mismatch between how educators describe their use of specific EBPs compared to how those EBPs are defined in the literature. Although many educators were able to describe how they used specific EBPs in their classroom with autistic children, they could not name that EBP nor did they describe their use of it in a way that matched the research description. These findings highlight the discrepancy between the terminology used in autism research and educators' implementation—researchers may define practices in ways that educators may not understand or apply in practice. There is a need for future research to document educators' specific adaptations to EBPs from research trials that may improve EBP fit to the local contexts and resources of schools.

## Limitations and future directions

We note several limitations, including participant characterization, homogeneity, and reliance on self-rated assessments. The representativeness of our sample on certain school- and district-wide characteristics was very high for Washington State and high for the United States. This adds inferential power to these findings as generalizable. However, there may be other characteristics for which we do not have important data, such as the proportion of autistic students, rates of autism training and awareness, and availability of funding for special education services. Second, although participants were a representative sample of WA and the USA, included educators were predominately white (86.2%). While a large percentage of general education (81.9%) and special education teachers in the United State identify as white (82.1%), there are significant differences in diversity across regions, type of district and schools (63), which may limit generalization of the present findings to other US and international school contexts. Third, data were collected during the 2020–2021 school year when schools were closed due to COVID-19, and we were unable to conduct live observations to corroborate EBP use with fidelity. Although necessary, reliance on self-report to understand EBP use is prone to response bias. To better understand EBP use to support inclusion and retention of autistic students, future studies should explore characterization factors, include a more diverse sample, and corroborate self-report data with direct observation of EBP use.

## Conclusions

In sum, the present paper highlights important overlap and divergence between how general and special educators and paraeducators are familiar, trained in, and use EBPs to support autistic students in inclusive education settings. Educators reported familiarity (98.8%), use (97.7%), and training (83.7%) in reinforcement and the least familiarity with behavioral momentum (29.1%), training in both video modeling and peer-mediated instruction and intervention (18.6%), and use of video modeling (14.0%). School personnel may benefit from more exposure, training, and collaboration related to what EBPs are, autism-specific EBPs, and selection of EBPs across education contexts. Due to increasing efforts to improve EBP use and inclusion of autistic students, public schools must consider how to better and more consistently train their educators to use EBPs to support their students with varying needs. These findings underscore the need for further research on how educators use EBPs as well as ways in which implementation supports may facilitate EBP use to support the inclusion and retention of autistic students.

## Data availability statement

The raw data supporting the conclusions of this article will be made available by the authors, without undue reservation.

## Ethics statement

The studies involving human participants were reviewed and approved by the University of Washington IRB (IRB ID: STUDY00010045) as well as each participating school district's Institutional Review Boards have approved the study procedures. The patients/participants provided their written informed consent to participate in this study.

## Author contributions

JL is the principal investigator of the study, generated the idea and designed the study, is the primary writer of the manuscript, and approved all changes. MDP is the study methodologist and drafted the data analysis sections. AH, MJ, and MH coded all qualitative data. AB and AO supported the writing of the manuscript. All authors were involved in developing, editing, reviewing, and providing feedback for this manuscript and have given approval of the final version to be published.

## Funding

This study was previously reviewed and approved and is funded by the Institute of Education Sciences [grant #: R324A200033 (JL)] in the amount of $1,399,375 and Institute of Education Sciences [grant #: #R305B170021 (Bruns)] in the amount of $702,476. The funder had no role in the design of this project, in the writing of the manuscript, and in the decision to submit this manuscript for publication.

## Conflict of interest

The authors declare that the research was conducted in the absence of any commercial or financial relationships that could be construed as a potential conflict of interest.

## Publisher's note

All claims expressed in this article are solely those of the authors and do not necessarily represent those of their affiliated organizations, or those of the publisher, the editors and the reviewers. Any product that may be evaluated in this article, or claim that may be made by its manufacturer, is not guaranteed or endorsed by the publisher.
